# Auditory discrimination and frequency modulation learning in schizophrenia patients: amphetamine within-subject dose response and time course

**DOI:** 10.1017/S0033291721001239

**Published:** 2023-01

**Authors:** Neal R. Swerdlow, Savita G. Bhakta, Jo Talledo, Lindsay Benster, Juliana Kotz, Sophia Vinogradov, Juan L. Molina, Gregory A. Light

**Affiliations:** 1 Department of Psychiatry, School of Medicine, University of California, San Diego, USA; 2 Department of Psychiatry, School of Medicine, University of Minnesota, USA; 3 VISN-22 Mental Illness Research Education and Clinical Center, VA San Diego Healthcare System, San Diego, CA, USA

**Keywords:** Amphetamine, auditory processing, cognitive training, neurocognition, schizophrenia

## Abstract

**Background:**

Auditory frequency modulation learning (‘auditory learning’) is a key component of targeted cognitive training (TCT) for schizophrenia. TCT can be effective in enhancing neurocognition and function in schizophrenia, but such gains require significant time and effort and elude many patients.

**Methods:**

As a strategy to increase and/or accelerate TCT-induced clinical gains, we tested the dose- and time-course effects of the pro-attentional drug, amphetamine (AMPH; placebo, 2.5, 5 or 10 mg po; within-subject double-blind, order balanced) on auditory learning in schizophrenia patients [*n* = 32; M:F = 19:13; age 42.0 years (24–55)]. To understand predictors and/or mechanisms of AMPH-enhanced TCT, we also measured auditory fidelity (words-in-noise (WIN), quick speech-in-noise (QuickSIN)) and neurocognition (MATRICS comprehensive cognitive battery (MCCB)). Some measures were also acquired from age-matched healthy subjects (drug free; *n* = 10; M:F = 5:5).

**Results:**

Patients exhibited expected deficits in neurocognition. WIN and QuickSIN performance at low signal intensities was impaired in patients with low *v.* high MCCB attention/vigilance (A/V) scores; these deficits were corrected by AMPH, maximally at 2.5–5 mg (*d*'s = 0.79–1.29). AMPH also enhanced auditory learning, with maximal effects at 5 mg (*d* = 0.93), and comparable effects 60 and 210 min post pill. ‘Pro-learning’ effects of AMPH and AMPH-induced gains in auditory fidelity were most evident in patients with low MCCB A/V scores.

**Conclusions:**

These findings advance our understanding of the impact of pro-attentional interventions on auditory information processing and suggest dose- and time-course parameters for studies that assess the ability of AMPH to enhance the clinical benefits of TCT in schizophrenia patients.

## Introduction

Patients with schizophrenia can benefit clinically from computerized targeted cognitive training (TCT) that utilizes a ‘bottom-up’ sensory training strategy. One form of TCT uses adaptive auditory frequency modulation exercises to enhance the accuracy and speed of auditory information processing. Conceptually, by improving neuronal responses to auditory stimuli, ‘bottom-up’ sensory training exercises produce gains in higher-order cognitive functions (Dale et al., 2016). As part of a larger suite of TCT exercises, after 30–40 h of training (over ~10 weeks) TCT is associated with significant and lasting neurocognitive gains in about half of schizophrenia patients (Adcock et al., 2009; Fisher, Holland, Subramaniam, & Vinogradov, 2010).

We are studying the pharmacological augmentation of cognitive training (‘PACT’), as a means to increase and/or accelerate TCT-induced clinical gains in schizophrenia patients (Swerdlow, 2011, 2012). Our working hypothesis is that neurocognitive and clinical gains from TCT result from engagement with and learning from the TCT exercises (Biagianti, Fisher, Neilands, Loewy, & Vinogradov, 2016), and therefore these gains will be enhanced and/or accelerated by interventions that enhance TCT engagement and learning. Because we know that attentional deficits impede engagement and learning, we specifically hypothesize that interventions that enhance attention in patients with attentional deficits will also enhance gains from TCT. This is the essence of the ‘PACT’ strategy: to amplify the benefits of cognitive training by targeting the neurocognitive domains essential for learning from that training. In a previous study (Swerdlow et al., 2017) in antipsychotic-medicated schizophrenia outpatients, a single 10 mg pill of the pro-attentional drug, d-amphetamine (AMPH), significantly enhanced learning of the frequency discrimination (‘Sound Sweeps’) component of TCT; this enhanced learning was retained when subjects were retested 7d later without AMPH.

In order to optimize treatment parameters for this PACT strategy, we conducted a within-subject dose–response and time-course study of AMPH effects on TCT in schizophrenia patients. In these same patients, we also assessed the effects of AMPH on auditory fidelity, as measured by performance on tasks of speech detection over masking backgrounds, and the relationship of those effects to both attentional capacity and the TCT-augmenting properties of AMPH. Based on past findings, we predicted that patients with the lowest attentional capacity would be most impaired in measures of auditory fidelity and learning, and would be most sensitive to the performance-enhancing effects of AMPH. We viewed the optimal dose and timing for AMPH effects as empirical questions.

## Methods

### Participants

Antipsychotic-medicated (stable regimen >30 days) patients with a primary diagnosis of schizophrenia or schizoaffective disorder (depressed type) were phone- or field-screened via a medical, psychiatric and substance history. After consent, qualifying patients came to the laboratory in <7 days for a ‘screen day’: a diagnostic assessment (M.I.N.I. 6.0; Sheehan et al., 1998), physical examination, electrocardiogram, vision and hearing tests, urine toxicology and pregnancy test. Eligible subjects (online Supplementary Table S1) completed measures of symptoms, ‘Sound Sweeps’ frequency discrimination threshold and the MATRICS comprehensive cognitive battery (MCCB) (Nuechterlein et al., 2008). Methods for the administration of the MCCB are found in online ‘Supplementary Methods’; continuous performance task (CPT) data were unavailable for two subjects. Patients were randomized to dose order (0, 2.5, 5, 10 mg po) and then tested four times at approximately weekly intervals, with TCT testing either 60 (*n* = 14) or 210 min (*n* = 18) post-pill. In addition to the measures collected on screen days, test days included measures of auditory fidelity (words-in-noise (WIN), quick speech-in-noise (QuickSIN)). Healthy subjects (*n* = 10) underwent screen day measures.

### Auditory fidelity testing

Auditory fidelity was tested using WIN (WIN; NIH Toolbox [Zecker et al., 2013]) and QuickSIN (QuickSIN; Etymotic Research, Elk Grove, IL [Killion, Niquette, Gudmundsen, Revit, & Banerjee, 2004]) modified to allow for binaural presentation. Detailed analyses of these measures are found in Bellis and Bellis (2015) and Sharma, Tripathy, and Saxena (2017). Both WIN and QuickSIN assess the ability to recognize speech over background noise (deciphering conversation in a noisy environment) and are deficient in schizophrenia patients (Iliadou et al., 2013; Ramage et al., 2016). Speech stimuli are presented in varying intensities of background conversation noise (four-talker babble); WIN uses one-word stimuli whereas QuickSIN utilizes sentence stimuli. Subjects repeat the words or sentences aloud, and responses are scored based on repetition accuracy of the word (WIN) or five ‘key’ words (QuickSIN). The primary measure for both is the # correct scores at each background dB level, with a maximum score of 5. These tests are widely used in audiologic assessments; ceiling effects in normal hearing subjects are expected when stimuli are >10 dB above the background noise and floor effects are expected when the words are presented 0 dB above background.

### Cognitive training

‘Sound Sweeps’ TCT (PositScience; brainhq.com; San Francisco, CA) is an auditory learning task based on frequency discrimination time-order judgment. Participants listened to a series of two successive tone sweeps (varying in frequency range and interstimulus interval [ISI]), and then indicated with two corresponding button presses whether the frequency increased or decreased within each tone, respectively (Fisher, Holland, Merzenich, & Vinogradov, 2009). The training is continuously adaptive – sweep duration, frequency range, and interstimulus interval become shorter after correct responses, but longer after incorrect responses. Baseline and best auditory processing speed (APS) scores are automatically calculated, with possible scores ranging from 16 to 1000 ms and lower scores indicating a better APS. On screen and 4 test days, subjects completed 1 h of TCT, as described in Swerdlow et al. (2017). These 5 h were the only TCT received in this study: 1 h on screen day, and 1 h on each of the 4 test days on which a pill was administered. A research assistant monitored each session. Analytic software yielded the key dependent measure for this study, which is the difference between the baseline (first) APS and the best of the subsequent trials; this serves as the operational measure of ‘APS learning’ (ms). Sound Sweeps training progresses through multiple ‘stages’, each of which was divided into three blocks; because many subjects completed only one stage during the 1 h session, analyses of APS learning included only that first stage. Three subjects exhibited ‘ceiling’ (1000 ms) latency scores both pre- and post-Sound Sweeps training, for both placebo and AMPH conditions, and key ‘AMPH-enhanced learning’ metrics are reported both with and without those subjects included.

### Timing

Relative to pill administration (*t* = 0), key measures were administered at 60 or 210 min (TCT) and at 275 or 280 min (WIN and QuickSIN, in that order).

Autonomic function and self-rating scales: Autonomic function (heart rate, blood pressure) and self-assessed levels of ‘happy’, ‘drowsy’, ‘focus attention’ and ‘anxious’ (100 mm visual analog scales (VAS)) were recorded at seven time points distributed across test days. Fewer than 0.05% of the ~2300 autonomic and 9200 VAS measures were unavailable and replaced by interpolating (averaging) temporally surrounding values. Autonomic data from the schizophrenia subjects had previously been included in a larger database reported in a study of potential adverse effects of AMPH in antipsychotic-medicated schizophrenia patients (Swerdlow et al., 2019).

### Data analyses

Primary dependent measures (learning [ms] change in APS; number of correct responses in WIN and QuickSIN) were analyzed by ANOVA with AMPH dose as a within-subject factor; models for WIN and QuickSIN included dB salience as a within-subject factor. Post-hoc contrasts utilized Fisher's least significant difference method. Primary, hypothesis-driven analyses are described in Results; complete ANOVAs are reported in online ‘Supplementary Results.’ Analyses of WIN and QuickSIN started with ANOVA's of placebo-dose performance, to test the hypotheses [based on (Swerdlow et al., 2020)] that (1) performance would decline sharply at ‘threshold’ salience levels of 4 and 5 dB in patients with attentional deficits, and that (2) threshold-level performance in attentionally-impaired subjects would be most sensitive to AMPH. For analyses of attention-dependence, MCCB A/V scores were divided into terciles [*n* = 10/group; CPT results were unavailable for two subjects)]. Analyses then assessed AMPH sensitivity of WIN and QuickSIN, to test the hypothesis that AMPH would enhance threshold-level performance, specifically among subjects with attentional deficits. QuickSIN measures include three separate speech ‘lists’; for this study, ‘List 1’ speech was used as a primary measure. Due to the limited range of scores (1–5) for WIN and QuickSIN, non-parametric analyses (Wilcoxon Signed Rank Test, Mann-Whitney *U* Test) were used to confirm significant effects detected by ANOVA. Analyses of TCT learning and its drug sensitivity were conducted via ANOVA (Swerdlow et al., 2017, 2020; see online ‘Supplementary Methods’); the key performance measure is APS in ms. This measure is analogous to a latency, i.e. slow ‘speed’ is indicated by a large APS. We predicted that AMPH would augment TCT learning (Swerdlow et al., 2017), particularly among subjects who are most deficient in attention. Consideration of potential contributions of age, smoking, antipsychotic dose (chlorpromazine equivalents) and anticholinergic burden (Campbell et al., 2016; Chew et al., 2008; Joshi et al., 2021; Vinogradov et al., 2009) to the main findings is reported in online ‘Supplementary Results’. Alpha was 0.05.

## Results

### Subject characteristics

Demographics and clinical characteristics of schizophrenia subjects (*n* = 32) and age-matched healthy subjects (*n* = 10) are shown in Table 1. Compared to healthy subjects, schizophrenia subjects were more likely to be smokers and less educated; schizophrenia subjects were deficient in MCCB performance (available in 30 schizophrenia subjects; MCCB composite T-score, the main effect of diagnosis: *F* = 15.89, df 1,38, *p* < 0.0005), with significant deficits in the speed of processing (*p* < 0.0005), attention/vigilance (*p* < 0.001), working memory (*p* < 0.005), visual learning (*p* < 0.0001) and social cognition (*p* < 0.02). As a group, the schizophrenia subjects were functionally impaired (GAF mean = 57.0), chronically ill (mean duration = 23.7 years; mean age of onset = 18.3 years), of below-average intelligence (mean WRAT = 91) and robustly medicated (mean chlorpromazine [CPZ] equivalent = 735 mg/day). Thirty-one subjects were taking second-generation antipsychotics; this included seven subjects taking clozaril and three taking aripiprazole. A full list of a psychoactive medication is shown in Table 1.

**Table 1.** Subject characteristics

	Schizophrenia	Healthy subjects	
Age (mean year, range)	42.1 (24–55)	41.1 (21–53)	
Sex (M:F)	19:13	5:5	
Smokers (%)	53.1	10.0	*p* < 0.017
Education (mean year, range)	12.1 (9–18)	15.1 (12–18)	*p* < 0.0001
MCCB T-scores [mean (s.e.m.s)]			
Speed of processing	34.7 (2.5)	53.5 (3.7)	*p* < 0.0005
Attention/vigilance	35.0 (2.9)	54.5 (3.1)	*p* < 0.001
Working memory	32.2 (2.4)	46.1 (3.0)	*p* < 0.005
Verbal learning	34.5 (1.8)	40.6 (3.5)	
Visual learning	36.3 (2.7)	57.2 (1.4)	*p* < 0.0001
Reasoning/problem solving	47.2 (2.2)	51.4 (2.7)	
Social cognition	34.1 (1.8)	43.8 (4.6)	*p* < 0.02
Duration Ill (mean year, range)	23.7 (7–42)		
GAF	57.0 (33–81)		
WRAT (mean (range))	91.0 (72–111)		
CPZ equivalents (mg/day (s.e.m.))	734.5 (136.1)		
Anticholinergic burden score^a^(mean (range))	4.97 (1–11)		
‘*N*’ taking regularly prescribed medications:			
First-generation antipsychotics	8		
Second-generation antipsychotics	31		
Clozapine	7		
Antidepressants	16		
Mood stabilizers	9		
Anxiolytics	2		
Anti-Parkinsonian drugs	11		
Anti-hypertensives	7		
Levothyroxine	3		

a Based on a summed total of 0–3 point scale for each medication, as described by Campbell et al. (2016), Chew et al. (2008) and Joshi et al. (2021) (see online Supplementary Table S3). GAF: Global Assessment of Functioning; WRAT: Wide Range Achievement Test (Wilkinson & Robertson, 2006).

### Autonomic and subjective effects

AMPH had minimal effects on subjective self-ratings and autonomic function as described in online ‘Supplementary Results’. The most robust evidence for AMPH bioactivity was seen in its modest positive chronotropic effects (online Supplementary Fig. 1).

### WIN and QuickSIN

**Fig. 1.**
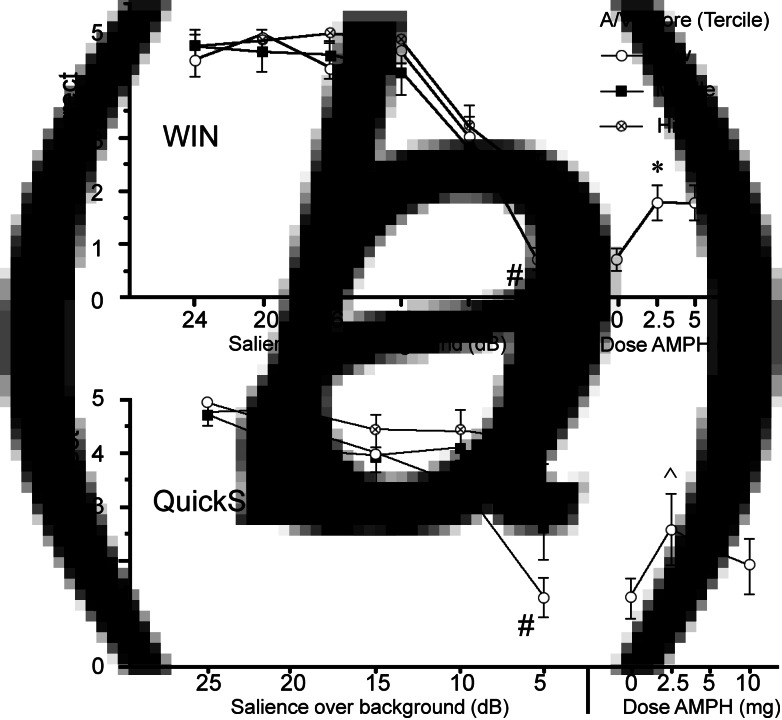
Correct identification of speech stimuli (out of five possible stimuli) after placebo in WIN (*a*) and QuickSIN (*b*) tests in schizophrenia subjects (*n* = 30) grouped in terciles based on MCCB A/V T-scores (*n* = 10/group). Stimuli with varying salience (WIN: 4–24 dB; QuickSIN: 5–25 dB) were superimposed over background noise. Compared to subjects with high and mid-level A/V scores, performance among low A/V subjects significantly deteriorated (#) when stimuli reached ‘thresholds’ of 4 dB (WIN) or 5 dB (QuickSIN) over a background. Plotted at right are gains in ‘threshold’ performance in this lowest A/V group after AMPH (WIN: 2.5–5 mg, *p's < 0.012, *d*'s = 1.29) or 5 dB (QuickSIN: 2.5 mg, ^ *p* < 0.06, *d* = 0.79); performance after AMPH in the lowest A/V subjects was comparable to what would be expected with an increase in stimulus salience by 3.14 dB (WIN) and 3.82 dB (QuickSIN) (see text). Full graphs are shown in the Supplementary Results.

Analyses of auditory fidelity measures first examined the performance under placebo conditions to assess the predicted ‘threshold’ levels of stimulus salience among subjects with poor attention. Subjects (*n* = 30) were ranked by A/V T-score and divided into terciles (*n* = 10/group). Threshold-levels of performance for WIN and QuickSIN were evident at 4 and 5 dB salience levels, respectively, seen as sharp declines in speech discrimination among the lowest-attention subjects compared to subjects with higher attentional capacity (Fig. 1*a*, *b*). For WIN, ANOVA confirmed a significant interaction of intensity (dB) × attention (*F* = 2.09, df 12, 162, *p* < 0.02); post-hoc comparisons confirmed impaired performance among the lowest-attention subjects at the 4 dB level (Fig. 1*a*). For QuickSIN, ANOVA confirmed a significant interaction of intensity (dB) × attention (*F* = 4.20, df 10, 135, *p* < 0.0001); post-hoc comparisons confirmed impaired performance among the lowest-attention subjects at the 5 dB level (Fig. 1*b*).

Analyses of WIN and QuickSIN that included active doses of AMPH (Fig. 1*a*, *b* and online Supplementary) suggested that AMPH predominantly enhanced ‘threshold’ performance among subjects with low MCCB A/V scores. For the inclusive group (*n* = 32), analyses revealed no significant main effects of AMPH on either WIN or QuickSIN performance (WIN: *F* = 1.97, df 3, 93, ns; QuickSIN: *F* < 1), and no interactions of AMPH × intensity (dB) (WIN: *F* < 1; QuickSIN: *F* = 1.35, df 12, 372, ns). For both measures, among subjects with the lowest A/V scores, AMPH exhibited ‘inverted-U’ dose effects. For WIN, compared to placebo, AMPH enhanced 4 dB performance at 2.5 and 5 mg doses (*p*'s < 0.012 and *d* = 1.29 for each), and this effect approached significance for 10 mg AMPH (*p* < 0.06; *d* = 0.98); these results were confirmed using non-parametric analyses (Wilcoxon signed-rank test: *p* < 0.016, 0.014 and 0.085 for 2.5, 5 and 10 mg AMPH *v.* placebo, respectively). For QuickSIN, AMPH-enhanced performance approached significance for the 2.5 mg dose (*p* < 0.06; *d* = 0.79); non-parametric comparisons also detected a near-significant effect at the 5 mg dose (*p* < 0.054). At these ‘threshold’ intensities, slopes of placebo performance functions for low A/V subjects were −0.35 words/dB (WIN) and −0.34 words/dB (QuickSIN); mean performance gains with AMPH in low A/V subjects (WIN: + 1.1 correct after 2.5 or 5 mg; QuickSIN: + 1.3 correct after 2.5 mg) corresponded to an effective increase in stimulus salience of 3.14 dB for WIN, and 3.82 dB for QuickSIN. AMPH raised performance in both WIN and QuickSIN among subjects with lowest A/V scores up to placebo-level scores in subjects with A/V scores in the middle and highest terciles (WIN) or the middle tercile (QuickSIN). To further demonstrate that AMPH-induced performance gains were greater among subjects with low *v.* high A/V scores, the magnitude of the ‘AMPH effect’ was calculated for each active dose based on a difference score (AMPH minus placebo). For both WIN and QuickSIN, this ‘AMPH effect’ was more robust among patients with the lowest *v.* highest tercile A/V T-scores (WIN: *F* = 4.38, df 1,18, *p* = 0.05; QuickSIN: *F* = 5.48, df 1,18, *p* < 0.035); this was confirmed via non-parametric comparisons for both WIN (5 mg: *p* < 0.03) and QuickSIN (5 mg: *p* < 0.04).

### Auditory processing speed

**Fig. 2.**
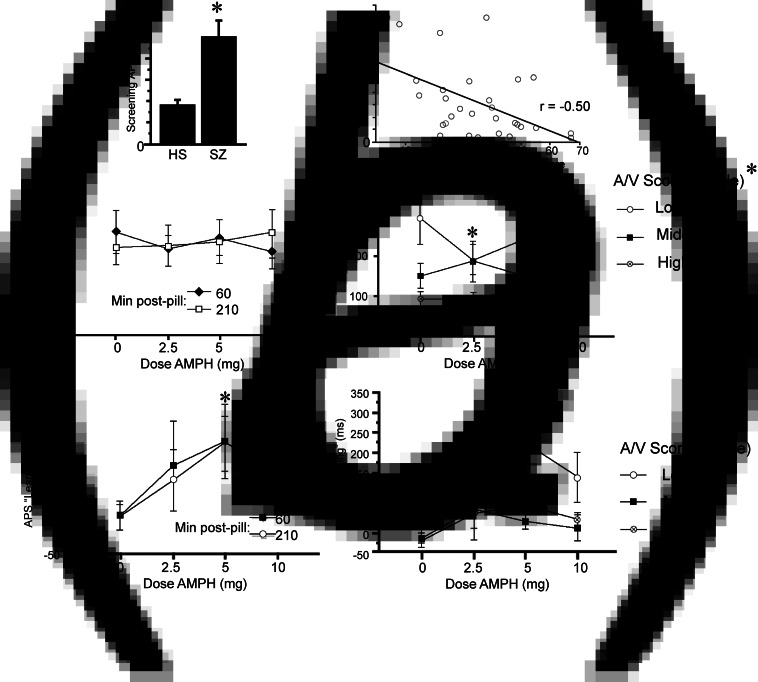
APS and APS learning during Sound Sweeps testing on screen day (*a*) and test days (*b* and *c*). (*a*) Baseline APS on screen day is significantly slowed in schizophrenia subjects *v.* healthy subjects (*p < 0.016). At right, APS in schizophrenia patients correlates significantly with A/V T-score (*p* < 0.005): shorter latencies (i.e. faster processing speed) were associated with greater A/V T-scores. Comparable results were obtained using log-transformed APS values (*p* < 0.005) or non-parametric statistics (*R*_s_ = −0.45, *p* < 0.015). (*b*) APS on test days was not impacted significantly by AMPH dose when analyzed across all subjects; at right, test day APS after placebo is significantly slower among schizophrenia subjects with the lowest A/V T-scores (*p* < 0.03 and <0.003 *v.* middle and high tercile groups, respectively); in the lowest A/V subjects, AMPH significantly enhanced APS (main effect: *p* < 0.0008; *p* < 0.0001, 0.035 and 0.002 for 2.5, 5 and 10 mg doses, respectively). (*c*) APS learning on test days was significantly enhanced by AMPH (*5 mg: *p* < 0.003; *d* = 0.93). At right, AMPH-enhanced APS learning was evident only among subjects with the lowest A/V T-scores (*5 mg: *p* < 0.02).

ANOVA of Sound Sweeps baseline performance on the screen day revealed slower APS values in patients *v.* HS (*F* = 6.41, df 1,37, *p* < 0.016; Fig. 2*a*). Among patients, slower APS was associated with lower A/V T-scores (*r* = −0.50, *p* < 0.005; Fig. 2*a*), consistent with Tarasenko et al. (2016). ANOVA of APS on test days during stage 1 (when learning was assessed) revealed no significant effect of AMPH dose (*F* < 1) or time (*F* < 1), and no dose × time interaction (*F* = 1.25, df 3,81, ns) (Fig. 2*b*). Data were collapsed across the two time points and examined for the interaction of AMPH dose with subject A/V scores. As it was on screen day, baseline APS on test days was inversely related with A/V T-score (*r* = −0.39, *p* < 0.045), i.e. the poorest attention was associated with the slowest APS. ANOVA with AMPH dose as a within-factor and A/V tercile as a between-factor confirmed the lack of the main effect of AMPH dose (*F* < 1); the main effect of A/V tercile did not reach significance (*F* = 2.71, df 2,24, *p* < 0.09), but there was a significant interaction of AMPH dose × tercile (*F* = 2.87, df 6,72, *p* < 0.015). Post-hoc analyses revealed that APS was significantly slowed among lowest-tercile subjects after placebo (*p* < 0.03 and 0.003 *v.* middle and highest tercile, respectively), and that AMPH significantly enhanced APS in (only) the lowest A/V tercile subjects (*F* = 8.00, df 3,24, *p* < 0.0008; *p* < 0.0001, 0.035 and 0.002 for 2.5, 5 and 10 mg doses, respectively).

### APS learning

Analysis of APS learning (ms) on the screen day detected no significant main effect of diagnosis (*F* < 1). Analysis of APS learning on test days (Fig. 2*c*) detected a significant main effect of AMPH dose (*F* = 2.89, df 3,81, *p* = 0.04) but not time (*F* < 1), and no dose × time interaction (*F* < 1). A comparable outcome was evident when analyses included 3 subjects who were TCT ‘non-learners’ (see ‘Methods’ section; AMPH dose: *F* = 2.87, df 3,90, *p* = 0.04; time: *F* < 1; dose × time: *F* = 1.05, df 3,90, ns). AMPH exhibited inverted-U dose effects, with maximal pro-learning effects at the 5 mg dose (*p* < 0.003; *d* = 0.93), and relatively weaker effects at 2.5 and 10 mg doses (*p*'s < 0.10 and 0.08, respectively; *d*'s = 0.45 and 0.62, respectively). Data were collapsed across the two time points and examined for the interaction of AMPH dose with subject A/V scores. ANOVA with AMPH dose as a within-factor and A/V tercile as a between-factor confirmed the main effect of dose (*F* = 3.28, df 3,72, *p* < 0.03) but not tercile (*F* = 2.79, df 2,24, *p* < 0.085) or an interaction of dose × tercile (*F* = 1.04, df 6,72, ns). Post-hoc comparisons revealed significant pro-learning effects of AMPH only among subjects in the lowest tercile of A/V scores (5 mg: *p* < 0.02). Despite the fact that A/V score strongly impacted APS in placebo-treated subjects (Fig. 2*b*), there was no apparent impact of A/V score on APS *learning* in placebo-treated subjects (Fig. 2*c*, right); the insensitivity of APS learning during 1 h of training to attentional capacity may reflect a ‘floor effect’, since there was near-zero learning in all schizophrenia subjects after placebo (Fig. 2c, left).

It is possible that AMPH-induced gains in APS (Fig. 2*b*) could interact with AMPH-induced gains in APS learning (Fig. 2*c*). For example, by increasing baseline APS (reducing ms latency), AMPH could compress the total ‘range’ available for APS learning, and this might artificially blunt the magnitude of APS learning. To examine the relationship between AMPH effects on baseline APS and APS learning, we assessed correlations between the magnitude of the ‘AMPH effect’ (active dose minus placebo) on APS baseline *v.* APS learning at each dose. Regression analyses revealed no significant correlation between AMPH-enhanced APS and AMPH-enhanced learning at 2.5 mg (*r* = −0.10, ns), 5 mg (*r* = 0.11, ns) or 10 mg doses (*r* = 0.10, ns). Thus, it is not likely that AMPH-induced changes in APS are significantly impacting AMPH-induced changes in APS learning.

## Discussion

We previously reported that 10 mg AMPH po administered 210 min prior to ‘Sound Sweeps’ training acutely enhanced TCT learning in antipsychotic-medicated schizophrenia patients and healthy subjects (Swerdlow et al., 2017). The magnitude of that previous AMPH effect (*d* = 0.62) was comparable to the magnitude of the effect detected for 10 mg AMPH in the present study (*d* = 0.56), not something we necessarily predicted based on the different study designs (one active dose and two tests *v.* three active doses and four tests). These earlier findings raised the possibility that AMPH might be used clinically to enhance the therapeutic impact of TCT in schizophrenia patients: TCT's therapeutic effects presumably reflect a learning process and an intervention that enhances TCT learning might thus be expected to improve (enhance, expedite or both) those therapeutic effects. We have also reported that attentional capacity is a strong determinant of TCT performance (Tarasenko et al., 2016), and that AMPH effects on both attention (Chou, Talledo, Lamb, Thompson, & Swerdlow, 2013) and on TCT learning (Swerdlow et al., 2017) are most robust among subjects with low baseline A/V T-scores; others (Biagianti et al., 2016) have reported that early engagement with TCT exercises strongly predicts TCT-associated neurocognitive gains. The present study was conducted to extend our past findings of AMPH-enhanced TCT by optimizing AMPH dose- and temporal parameters and to begin to explore mechanisms that might contribute to this AMPH-enhanced TCT learning. The apparent ‘inverted-U’ dose function identified in AMPH performance-enhancing effects in WIN, QuickSIN and TCT is generally consistent with a large literature reporting inverted-U dose functions for psychostimulant effects on a long list of behavioral measures across species (cf. Lyon & Robbins, 1975; Robbins & Sahakian, 1979).

The long-term goal of this type of inquiry is to inform a clinical trial assessing the feasibility and efficacy of using drugs with pro-learning effects to enhance the therapeutic gains from TCT; in one model, combined use of medications and TCT could be accomplished in a controlled outpatient setting that allows for careful monitoring of medication and its controlled delivery in concert with TCT. The present findings confirm that AMPH can enhance TCT learning in antipsychotic-medicated schizophrenia patients; these effects were most robust with 5 mg, and were comparable in magnitude when learning was assessed 60 and 210 min post-pill. We do not believe that 5 mg will be the optimal ‘pro-learning’ dose of AMPH for *all* schizophrenia patients; in fact, our present findings suggest that *this dose might only have ‘pro-learning’ effects among patients with attentional deficits*. Still, to the degree that these pro-learning effects – extended over a full course of 30–40 TCT sessions – might augment TCT-associated therapeutic gains, the present findings suggest that such benefits among attentionally impaired schizophrenia patients might be achieved with a relatively low dose of AMPH (comparable to a ‘starting dose’ used to treat attentional deficits in a young child [Smucker & Hedayat, 2001]) and with a time course that would be feasible for use in a controlled outpatient setting (TCT starting 60 min post pill). The identification of a treatment-sensitive subgroup of patients within a highly heterogeneous clinical entity may be an important step towards a ‘personalized medicine’ approach to schizophrenia.

Pro-cognitive effects of AMPH in antipsychotic-medicated schizophrenia patients have been reported for many years (e.g. Barch & Carter, 2005; Goldberg, Bigelow, Weinberger, Daniel, & Kleinman, 1991; cf. Solmi *et al*., 2018). While the present results do not address the neural substrates responsible for AMPH pro-cognitive effects in schizophrenia patients, we (Swerdlow et al., 2018) and others (Goldberg et al., 1991) proposed that these AMPH effects may reflect the preferential activation of prefrontal D1-family receptors by AMPH-induced dopamine release under conditions of antipsychotic-induced D2-family blockade; we were able to reproduce related AMPH effects in rodent models under varying levels of D1- and D2 receptor blockade (Swerdlow et al., 2018). Consistent with models of a prefrontal D1-regulation of neurocognition and specifically attention (Arnsten, 1998), the observed effects of AMPH in our past (Swerdlow et al., 2017, 2018) and present studies interact with baseline neurocognitive characteristics of our schizophrenia subjects: subjects with the most impaired baseline A/V T-scores exhibited the greatest AMPH-induced gains in performance in several measures. Conceivably, impaired attention might reflect a neuropathological process (e.g. deficient dopamine activity at prefrontal D1 receptors) that could produce a state (e.g. ‘upregulated’ D1 receptors) that would be particularly sensitive to the effects of AMPH-induced dopamine release. Interestingly, the key measure in this study – auditory learning in a ‘Sound Sweeps’ frequency modulation task – is a form of sensory learning, thought to occur relatively early in auditory circuitry, far from the prefrontal cortex (Vinogradov, Fisher, & de Villers-Sidani, 2012). Our findings suggest that such ‘bottom-up’ learning can be enhanced via gains in ‘top-down’ attentional mechanisms; hypothetically, plasticity observed in thalamic structures after a full course of TCT might reflect the anatomical impact of this convergence of prefrontal and early sensory activity (Ramsay et al., 2018). At a more practical level, the most parsimonious interpretation of these findings may be that even early sensory learning is enhanced when subjects with impaired attention are better able to attend to the learning task.

One alternative explanation for the present findings is that AMPH may enhance TCT learning by reversing medication-induced learning deficits. Conceivably, both the anticholinergic and antidopaminergic effects of medications used by most patients in this study (Table 1) might impede auditory learning. Vinogradov et al. (2009) reported on the cognitive costs of anticholinergic burden (ACB) on the neurocognitive gains associated with TCT: serum anticholinergic activity was negatively correlated with these gains, and accounted uniquely for 20% of the variance in global cognitive change after TCT. In the present study, AMPH-induced gains in auditory learning were not associated with either ACB or chlorpromazine equivalents (Supplementary Table S3). While the present study did not assess clinical gains *per se*, it is interesting that two of the three ‘non-learner’ patients in this study had the first- and second-highest ACB scores (11 and 10), while the third ‘non-learner’ was in the lowest decile for WRAT scores (79 years) and highest decile for duration of illness (39 years). The pathway to both TCT learning and clinical sensitivity is certainly multifactorial, but our results are consistent with the notion that ACB may be one moderating factor.

The notion that auditory discrimination can be enhanced by pro-attentional effects of AMPH is consistent with our findings that schizophrenia patients with low attentional capacity are impaired in their ability to discriminate spoken words over a masking background (in WIN and QuickSIN) and that this ability can be significantly improved by low doses of AMPH (2.5–5 mg). This finding does not *directly* implicate pro-attentional effects of AMPH as the mechanism by which this drug enhances auditory learning and discrimination: it remains possible that these AMPH effects are mediated via more basic mechanisms – perhaps at the peripheral sensory level – that nonetheless have the greatest impact on performance in patients with the poorest attention. A previous report (Breitenstein et al., 2004) suggests that simple arousal cannot account for the ability of AMPH to enhance spoken word learning in healthy subjects, but that such pro-learning effects might be associated with the positive hedonic effects of this drug.

We discussed the potential risks of administering AMPH to antipsychotic-medicated schizophrenia patients in Swerdlow et al. (2019). That report analyzed the clinical impact of AMPH administration in a series of studies completed in our facility over a 4-year period; the cumulative ‘*n*’ of 53 patients included most of the participants of the present study. Our findings were clear: in antipsychotic-medicated schizophrenia patients, 1–3 doses of AMPH (cumulatively, 10–17.5 mg po) were not associated with detrimental subjective, autonomic, symptomatic or functional changes. Still, it is important to emphasize that *all evidence to-date from our studies was generated in patients taking robust regimens of antipsychotic medications*; this fact may account not only for the apparent lack of adverse events, but also *may be a direct causative factor in the mechanisms underlying AMPH-induced gains in our experimental measures*. Moreover, our findings do not fully address the risks of exposing antipsychotic-medicated schizophrenia patients to up to three 5 mg doses of AMPH per week for 10 weeks, as proposed in the PACT model for AMPH-enhanced TCT effects. However, evidence exists to this effect; for example, daily dosing of AMPH for 10 weeks at doses >5 mg/day in antipsychotic-medicated schizophrenia patients is associated with clinical gains but not adverse effects (Lasser et al., 2013). Conceivably, the use of long-acting injectable antipsychotics in a PACT model might be a practical way to ensure that AMPH exposure occurred exclusively in the context of adequate antipsychotic coverage.

We chose to pursue these studies with AMPH because it is perhaps the most-studied psychostimulant with pro-attentional properties in addition to its potentially ‘pro-neuroplasticity’ properties associated with enhanced auditory learning (Breitenstein et al., 2004). But clearly, drugs other than AMPH might be useful – and potentially preferable to AMPH – in a PACT therapeutic approach. For example, D-cycloserine was being reported to enhance TCT learning and reduce symptoms in patients with schizophrenia (compared to patients treated with placebo + TCT) (Cain et al., 2014). McClure et al. (2019) reported that pro-cognitive effects of computerized cognitive remediation (not TCT as reported here) were significantly enhanced by the alpha 2A-adrenergic agonist, guanfacine, in individuals with a schizotypal personality disorder. Lenze et al. (2020) reported that the putatively pro-cognitive and pro-neuroplastic drug, vortioxetine, boosted the global cognitive gains produced by computerized cognitive training in adults age 65 years and older with age-related cognitive decline. We previously reported that the NMDA antagonist, memantine, enhanced TCT learning and produced gains in measures of auditory discrimination (WIN, QuickSIN) in schizophrenia patients (Swerdlow et al., 2020). The characteristics of memantine effects on WIN performance (gains limited to a ‘dynamic range’ of 4–8 dB salience and most evident in patients with low A/V T-scores) are quite similar to those exhibited by AMPH in the present study.

There are clear limitations to this study. Participants did not receive a ‘full course’ of TCT: patients received only 5 total hours of TCT, 1 h on screen days and 1 h on each of the 4 test days, following pill administration. Thus, this experimental medicine design did not allow us to assess clinical gains from AMPH-enhanced learning. Furthermore, our previous findings (Swerdlow et al., 2017) suggested that AMPH-enhanced auditory learning ‘carried forward’ for a week, suggesting that learning during the four TCT sessions in this within-subject study might have been impacted by learning in previous weeks, and potentially by previous (randomized) AMPH dose exposure. Nonetheless, based on its ability to acutely enhance TCT learning in this study, we have initiated studies of the ability of AMPH to enhance and/or accelerate the clinical and neurocognitive gains produced by TCT (30 1 h sessions) in schizophrenia, and similar studies are scheduled to begin using memantine. In the case of AMPH, both dose (5 mg) and timing of auditory training (60 min post pill) were selected based on findings from the present study; our prediction is that AMPH will augment the therapeutic impact of TCT in these patients, and particularly among those with the lowest attentional capacity.

## Supplementary material

For supplementary material accompanying this paper visit https://doi.org/10.1017/S0033291721001239. click here to view supplementary material
