# Supplementary material for: Auditory discrimination and frequency modulation learning in schizophrenia patients: amphetamine within-subject dose response and time course
Source: Psychol Med. 2021 Apr 14;53(1):140–8. doi: 10.1017/S0033291721001239 (PMC8514598; doi:10.1017/S0033291721001239)
Supplement: Supplementary file 1 [file S0033291721001239sup001.docx]

**Supplemental Methods**

MCCB: The MCCB was developed to evaluate neurocognition in trials of procognitive therapies for SZ and is accepted by the FDA as a primary endpoint (Nuechterlein et al., 2008). The MCCB measures 7 key cognitive domains relevant to cognitive deficits in SZ and includes ten tests that assess: speed of processing (SP), attention/vigilance (AV), working memory (WM), verbal learning (VL), visual learning (VsL), reasoning and problem solving (RP), and social cognition (SC), and provides T-scores for each domain and a composite score of all domains. The MCCB was administered to healthy subjects (n=10) and schizophrenia subjects (n=32) on the Screen Day; equipment malfunction led to the loss of Continuous Performance Test data (and hence A/V scores) in two schizophrenia subjects.

**Supplemental Results**

Supplemental Figure 1 shows autonomic and subjective measures in schizophrenia subjects after AMPH. Arrows indicate two time points of TCT “Sound Sweeps” testing. The only modest effect of AMPH on these measures is seen in its dose-dependent increase in heart rate (difference from baseline), which reached only trend levels (F=2.41, df 3,93, p < 0.072; placebo vs. 10 mg dose: p<0.012 (*)). A minimal effect of AMPH on autonomic measures in antipsychotic-medicated patients has been reported previously by our group (Swerdlow et al. 2019), but was originally reported by Modell and Hussar (1965). When subjects were divided into terciles based on antipsychotic dosing (CPZ equivalents), ANOVAs of AMPH effects on heart rate, systolic and diastolic blood pressure all revealed no significant main effects of tercile (all F’s<1), and no significant interactions of tercile x time (data not shown).

Supplemental Figure 2-3 show the full dose-response functions for WIN (Figure S2) and QuickSIN (Figure S3) across all stimulus intensities, based on A/V scores (terciles, n=10/tercile). Encircled values show significant drop in performance (#) among the lowest A/V group at the “threshold” intensities (4 and 5 dB in WIN and QuickSIN, respectively). Asterisks indicate significant increase from placebo-level performance in this low A/V group after 2.5 or 5 mg AMPH (**, WIN) or 2.5 mg AMPH (*, QuickSIN).

Supplemental Figure 4 shows WIN and QuickSIN performance for HS (n=10) tested with no pill vs. schizophrenia (SZ) patients (n=32) tested with placebo dose. No group differences in correct responses, nor interactions of group x dB level, were detected (all F’s < 1). In interpreting these data it is important to note that HS completed WIN and QuickSIN only once, while the schizophrenia patients’ “placebo” level performance followed 1-3 tests with active AMPH doses. Thus, groups differ both by diagnosis (HS vs. schizophrenia) and by practice level (HS < schizophrenia).

Supplemental Table 1 lists study inclusion / exclusion criteria. Supplemental Table 2 provides full ANOVA values for WIN, QuickSIN, APS and APS learning; key contrasts are described in the main Results text. Supplemental Table 3 shows relationships between APS and APS learning and 5 variables: Age, smoking status, antipsychotic burden (chlorpromazine (CPZ) equivalents), anticholinergic burden (ACB) and Wide-Ranging Achievement Test (WRAT) scores (used as a proxy for pre-morbid IQ). ACB scores were assigned to each subject as the sum total of scores associated with all medications; ACB scores (0-3) for common medications are based on Chew et al. (2008), Campbell et al. (2016) and Joshi et al. (2021). No robust relationships were detected between these variables and AMPH sensitivity in measures of APS or APS learning at any dose of AMPH. Supplemental Table 4 lists full MCCB scores by A/V tercile, as well as potentially relevant correlations among clinical variables and A/V score. No significant correlations were detected between A/V score and age, education, duration of illness, CPZ equivalents or ACB score, but A/V score did correlate significantly with WRAT score (p<0.002). Importantly, when AMPH effect on TCT learning was examined based in WRAT tercile, ANOVA revealed no significant main effect of tercile (F=1.17, df 2,26, ns), a significant effect of AMPH dose (F=3.07, df 3,78, p<0.035) and no significant interaction of tercile x dose (F<1).

**References**

Campbell, N. L., Perkins, A. J., Bradt, P., Perk, S., Wielage, R. C., Boustani, M. A., & Ng, D. B. (2016). Association of anticholinergic burden with cognitive impairment and health care utilization among a diverse ambulatory older adult population. Pharmacotherapy, 36, 1123–1131. doi:10.1002/phar.1843.

Chew, M. L., Mulsant, B. H., Pollock, B. G., Lehman, M. E., Greenspan, A., & Mahmoud, R. A. … & Gharabawi, G. (2008). Anticholinergic activity of 107 medications commonly used by older adults. Journal of the American Geriatrics Society, 56, 1333–1341. doi:10.1111/j.1532-5415.2008.01737.x.

Joshi, Y. B, Thomas, M. L., Braff, D. L., Green, M. F., Gur, R. C., & Gur, R. E. … & Light, G.A. (2021). Comprehensive characterization of anticholinergic medication burden-associated cognitive impairment in schizophrenia. American Journal of

Psychiatry, (in press).

Modell, W., & Hussar, A. E. (1965). Failure of dextroamphetamine sulfate to influence eating and sleeping patterns in obese schizophrenic patients: Clinical and pharmacological significance. Journal of the American Medical Association (JAMA), 193, 275–278. doi:10.1001/jama.1965.03090040019006.

Nuechterlein, K. H., Green, M. F., Kern, R. S., Baade, L. E., Barch, D. M., & Cohen, J. D. … & Marder, S.R. (2008). The MATRICS consensus cognitive battery, part 1: Test selection, reliability, and validity. American Journal of Psychiatry, 165(2), 203–213. doi:10.1176/appi.ajp.2007.07010042.

Swerdlow, N. R., Bhakta, S. G., Talledo, J., Benster, L., Kotz, J., Lavadia, M., & Light, G. A. (2019). Lessons learned by giving amphetamine to antipsychotic-medicated schizophrenia patients. Neuropsychopharmacology, 44, 2277–2284. doi:10.1038/s41386-019-0495-4.
