# Supplementary material for: Auditory discrimination and frequency modulation learning in schizophrenia patients: amphetamine within-subject dose response and time course
Source: Psychol Med. Author manuscript; Available in PMC 2024 Jan 1. (PMC8514598; doi:10.1017/S0033291721001239)
Supplement: Supplementary file 1 [file NIHMS1701619-supplement-Supplemental_Methods__Tables___Figures.pdf]

Supplemental Methods

MCCB: The MCCB was developed to evaluate neurocognition in trials of procognitive therapies for SZ and is accepted by the FDA as a primary endpoint (Nuechterlein et al., 2008). The MCCB measures 7 key cognitive domains relevant to cognitive deficits in SZ and includes ten tests that assess: speed of processing (SP), attention/vigilance (AV), working memory (WM), verbal learning (VL), visual learning (VsL), reasoning and problem solving (RP), and social cognition (SC), and provides T-scores for each domain and a composite score of all domains. The MCCB was administered to healthy subjects (n=10) and schizophrenia subjects (n=32) on the Screen Day; equipment malfunction led to the loss of Continuous Performance Test data (and hence A/V scores) in two schizophrenia subjects.

Supplemental Results

Supplemental Figure 1 shows autonomic and subjective measures in schizophrenia subjects after AMPH. Arrows indicate two time points of TCT “Sound Sweeps” testing. The only modest effect of AMPH on these measures is seen in its dose-dependent increase in heart rate (difference from baseline), which reached only trend levels ($F=2.41$, df 3,93, $p < 0.072$; placebo vs. 10 mg dose: $p<0.012$ (*)). A minimal effect of AMPH on autonomic measures in antipsychotic-medicated patients has been reported previously by our group (Swerdlow et al. 2016, 2019), but was originally reported by Modell and Hussar (1965). When subjects were divided into terciles based on antipsychotic dosing (CPZ equivalents), ANOVAs of AMPH effects on heart rate, systolic and diastolic blood pressure all revealed no significant main effects of tercile (all F’s<1), and no significant interactions of tercile x time (data not shown).

Supplemental Figure 2-3 show the full dose-response functions for WIN (Figure S2) and QuickSIN (Figure S3) across all stimulus intensities, based on A/V scores (terciles, n=10/tercile). Encircled values show significant drop in performance (#) among the lowest A/V group at the “threshold” intensities (4 and

5 dB in WIN and QuickSIN, respectively). Asterisks indicate significant increase from placebo-level performance in this low A/V group after 2.5 or 5 mg AMPH (**, WIN) or 2.5 mg AMPH (*, QuickSIN).

Supplemental Figure 4 shows WIN and QuickSIN performance for HS (n=10) tested with no pill vs. schizophrenia (SZ) patients (n=32) tested with placebo dose. No group differences in correct responses, nor interactions of group x dB level, were detected (all F's < 1). In interpreting these data it is important to note that HS completed WIN and QuickSIN only once, while the schizophrenia patients' "placebo" level performance followed 1-3 tests with active AMPH doses. Thus, groups differ both by diagnosis (HS vs. schizophrenia) and by practice level (HS < schizophrenia).

Supplemental Table 1 lists study inclusion / exclusion criteria. Supplemental Table 2 provides full ANOVA values for WIN, QuickSIN, APS and APS learning; key contrasts are described in the main Results text. Supplemental Table 3 shows relationships between APS and APS learning and 5 variables: Age, smoking status, antipsychotic burden (chlorpromazine (CPZ) equivalents), anticholinergic burden (ACB) and Wide-Ranging Achievement Test (WRAT) scores (used as a proxy for pre-morbid IQ). ACB scores were assigned to each subject as the sum total of scores associated with all medications; ACB scores (0-3) for common medications are based on Chew et al. (2008), Campbell et al. (2016) and Joshi et al. (2021). No robust relationships were detected between these variables and AMPH sensitivity in measures of APS or APS learning at any dose of AMPH. Supplemental Table 4 lists full MCCB scores by A/V tercile, as well as potentially relevant correlations among clinical variables and A/V score. No significant correlations were detected between A/V score and age, education, duration of illness, CPZ equivalents or ACB score, but A/V score did correlate significantly with WRAT score (p<0.002). Importantly, when AMPH effect on TCT learning was examined based in WRAT tercile, ANOVA revealed no significant main effect of tercile (F=1.17, df 2,26, ns), a significant effect of AMPH dose (F=3.07, df 3,78, p<0.035) and no significant interaction of tercile x dose (F<1).

Table S1. Inclusion Criteria

For both SZ subjects and HS:

1. English speaking
2. 18-55 years of age
3. Able to provide written informed consent
4. No hearing deficits, color blindness, mental retardation or medical exclusion criteria (see text)
5. Heart rate 55 - 95, Systolic blood pressure 90 - 160, Diastolic blood pressure 45-95
6. UTox negative for recreational drugs
7. Not pregnant; double barrier contraception

For HS:

1. No current or past DSM IV TR Axis I diagnosis on SCID-NP

For SZ subjects:

1. Axis I diagnosis of SZ or schizoaffective disorder, depressed type on M.I.N.I. plus 6
2. No psychiatric hospitalization in the past 2 months
3. No history of substance dependence/abuse OR in full remission for more than 6 months at the time of screening
4. Stable antipsychotic regimen for past month
5. No history of TCT treatment

UTox: urine toxicology screen

Supplemental Table 2. ANOVAs for analyses of WIN, QuickSIN and TCT

	Factor	F	df	p	Post-hoc comparisons
**WIN**	Dose	1.50	3,81	ns	
	A/V (Tercile)	1.35	2,27	ns	
	Dose x Tercile	< 1			
	dB	148.79	5,135	<0.0001	
	dB x Tercile	1.21	10,135	ns	
	dB x Dose	< 1			
	dB x Dose x Tercile	1.66	30,405	<0.018	
WIN 4 dB					
Lowest Tercile	Dose				Placebo vs. 2.5 mg: p<0.012
					Placebo vs. 5 mg: p<0.012
					Placebo vs. 10 mg: p<0.06
WIN 4 dB "AMPH effect"					
Lowest vs. highest Tercile		4.38	1,18	= 0.05	Mann-Whitney U: U=22.5, p<0.028
**QuickSIN**					
	Dose	<1			
	A/V (Tercile)	4.25	2,27	<0.025	
	Dose x Tercile	1.05	ns		
	dB	50.67	4,108	<0.0001	
	dB x Tercile	5.24	8,108	<0.0001	
	dB x Dose	1.20	ns		
	dB x Dose x Tercile	1.48	24,324	<0.072	
QuickSIN 5 dB					
Lowest tercile	Dose				Placebo vs. 2.5 mg: p<0.06
QuickSIN 5 dB "AMPH effect"					
Lowest vs. highest Tercile		5.48	1,18	<0.035	Mann-Whitney U: U=23.5, p<0.04
**APS (Test Day)**					
	Dose	< 1			
	Tercile	2.71	2,24	<0.09	
	Dose x Tercile	2.87	6,72	<0.015	
Lowest Tercile	Dose				Placebo vs. 2.5 mg: p<0.0001
					Placebo vs. 5 mg: p=0.03
					Placebo vs. 10 mg: p<0.002
**APS "Learning"**					
	Dose	3.28	3,72	<0.026	Placebo vs. 5 mg: p<0.003
	Tercile	2.79	2,24	<0.082	
	Dose x Tercile	1.04	6,72	ns	
Lowest Tercile	Dose				Placebo vs. 5 mg: p<0.02

Supplemental Table 3. TCT performance vs. age, smoking status, medications and WRAT

	Age[1]	Smoke? (yes/no)[2]	CPZ equiv.[1]	ACB[2,3]	WRAT[1,4]
APS (placebo)	0.24	ns	0.00	ns	-0.32
AMPH effect on APS:					
2.5 mg	0.01	ns	0.21	ns	-0.36
5 mg	-0.02	ns	0.25	ns	-0.03
10 mg	-0.19	ns	0.25	ns	0.00
AMPH effect on learning:					
2.5 mg	0.22	ns	-0.16	ns	0.06
5 mg	0.23	ns	-0.10	ns	0.04
10 mg	0.39*	ns	-0.16	ns	-0.06

1. r value
2. 1-factor ANOVA
*p<0.04
3. Tercile split for anticholinergic burden (ACB), calculated as in Joshi et al. (2021). Based on known anticholinergic activity (e.g. Chew et al. (2008)), medications were assigned low (e.g. “0” for buspirone) or high (e.g. “3” for chlorpromazine) values on a 0-3 scale. Total ACB was calculated as the sum of these values for all routine medications for each patient, yielding a range of 0-11.
4. Wide-Range Achievement Test (Wilkinson and Robertson, 2006)

Supplemental Table 4. MCCB T-scores for patients, divided by A/V tercile (mean (SEM))

A/V Tercile*	SP	WM	VL	Vis L	RPS	SC
1 (17.9 (2.3))	22.6 (3.8)	19.6 (2.8)	26.2 (1.4)	26.6 (3.2)	39.6 (3.1)	27.7 (2.8)
2 (34.5 (1.6))	37.6 (2.9)	35.8 (2.5)	33.3 (1.7)	39.0 (5.0)	50.7 (2.7)	37.6 (2.8)
3 (52.7 (2.7))	43.8 (3.2)	41.1 (3.4)	44.1 (3.1)	43.3 (4.4)	51.4 (4.3)	37.0 (3.2)

SP: Speed of Processing; A/V: Attention / Vigilance; WM: Working Memory; VL: Verbal Learning; Vis L: Visual Learning; RPS: Reasoning/Problem Solving; SC: Social Cognition

* A/V score correlations (R): Age (y) = 0.03 (ns); duration ill (y) = -0.10 (ns); education (y) = 0.26 (ns); WRAT = 0.59 ($p<0.002$); CPZ equivalents = -0.23 (ns); ACB score = -0.12 (ns).

Fig. S1.

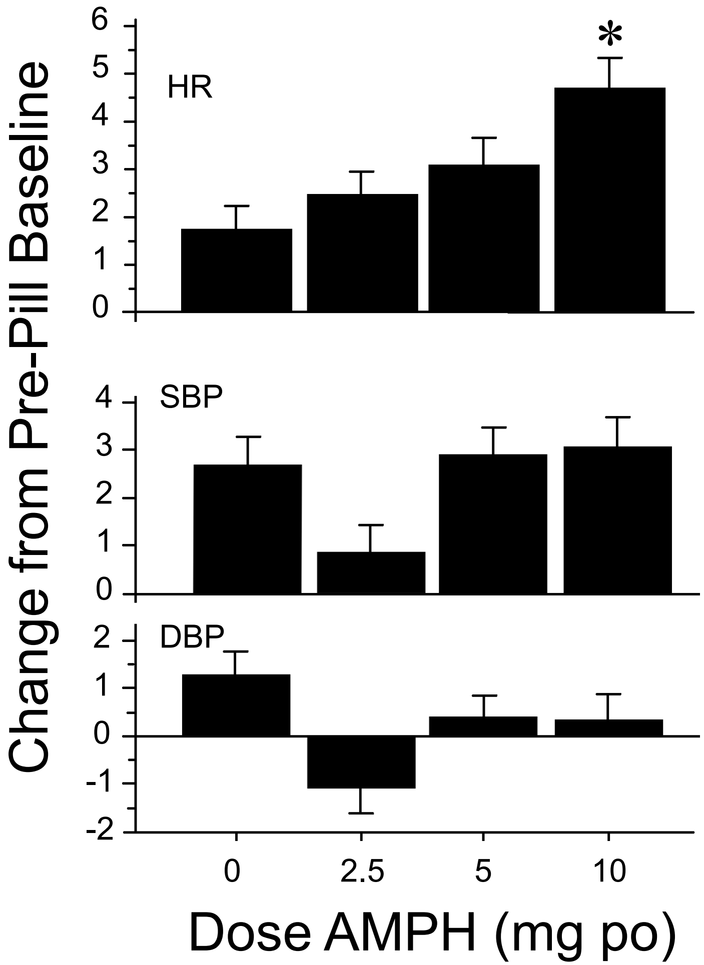

Change from Pre-Pill Baseline
HR
SBP
DBP
*
6
5
4
3
2
1
0
-1
-2
0
2.5
5
10
Dose AMPH (mg po)

Fig. S2.

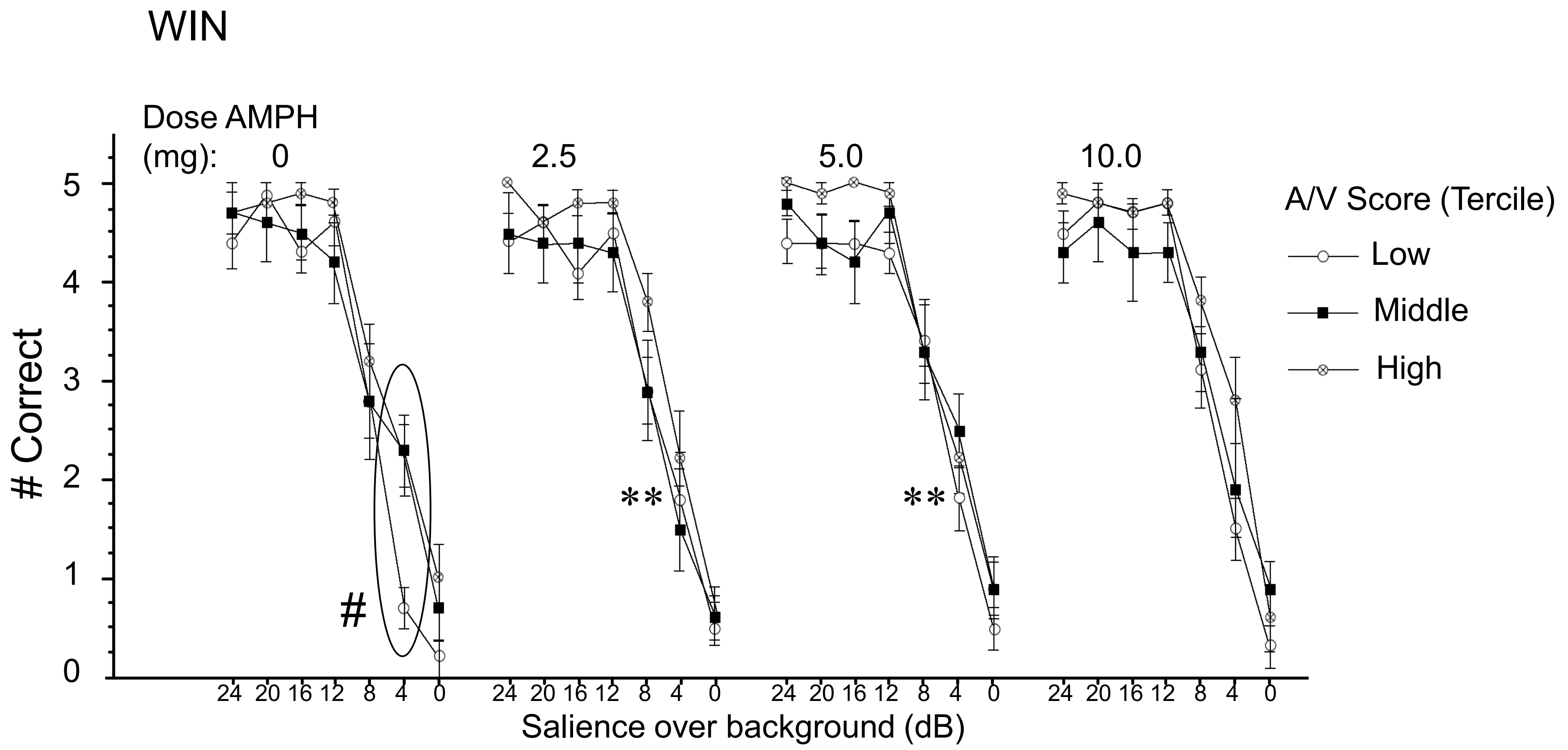
WIN
Dose AMPH
(mg): 0
2.5
5.0
10.0
Correct
0
1
2
3
4
5
#
**
**
24 20 16 12 8 4 0
Salience over background (dB)
A/V Score (Tercile)
Low
Middle
High

Fig. S3.

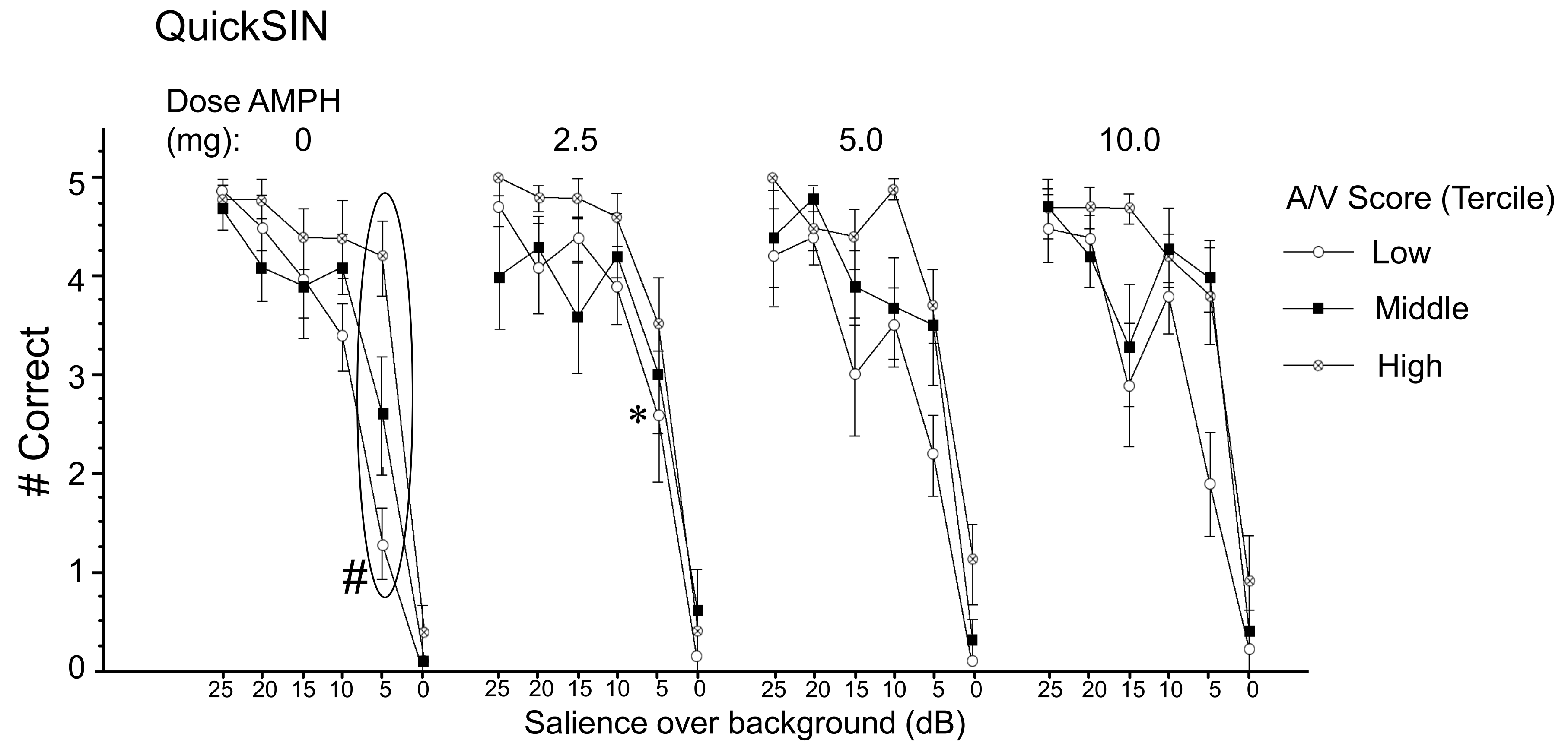
QuickSIN
Dose AMPH
(mg): 0
2.5
5.0
10.0
Correct
Salience over background (dB)
A/V Score (Tercile)
Low
Middle
High
#
*

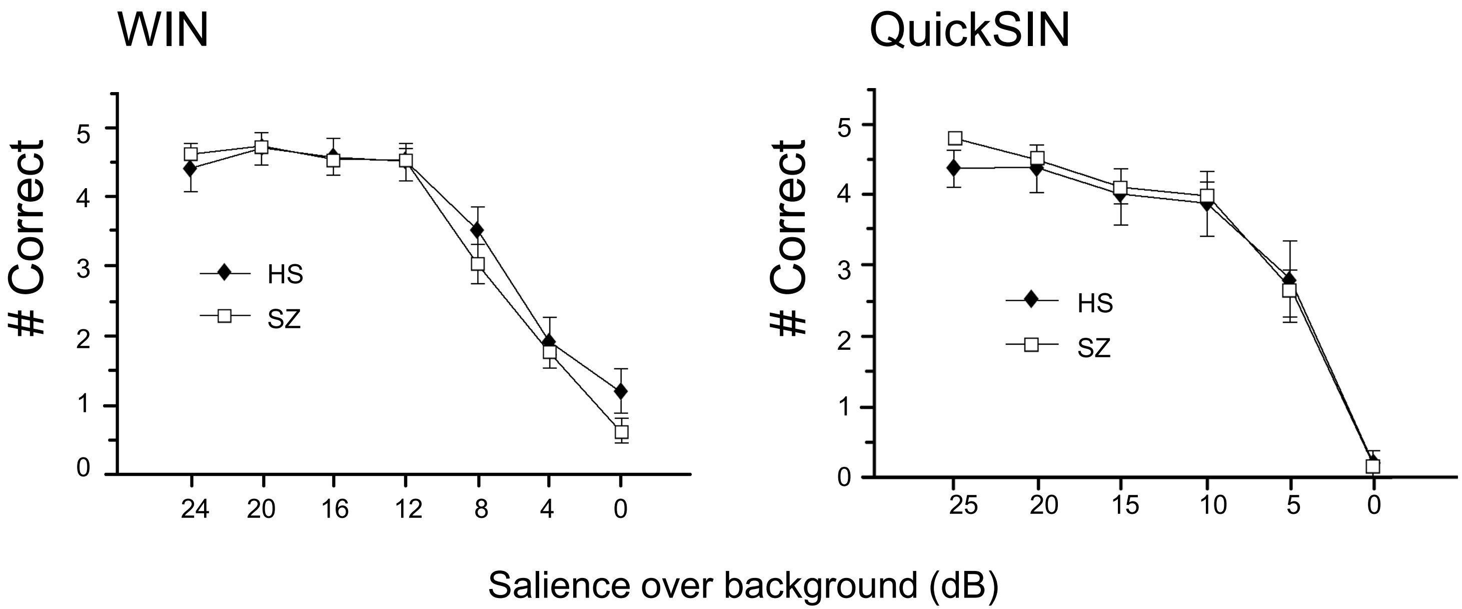
WIN
QuickSIN
Correct
HS
SZ
0
1
2
3
4
5
24
20
16
12
8
4
0
25
20
15
10
5
0
Salience over background (dB)
